# A New Spatane Diterpenoid from the Cultured Soft Coral *Sinularia leptoclados*

**DOI:** 10.3390/md11010114

**Published:** 2013-01-10

**Authors:** Tsung-Chang Tsai, Yu-Jen Wu, Jui-Hsin Su, Wei-Tung Lin, Yun-Sheng Lin

**Affiliations:** 1 Department of Superintendent, Antai Medical Care Cooperation Antai Tian-Sheng Memorial Hospital, Pingtung 92842, Taiwan; E-Mail: a088186@mail.tsmh.org.tw; 2 Department of Beauty Science, Meiho University, Pingtung 91202, Taiwan; E-Mail: wyr924@ms24.hinet.net or x00002180@meiho.edu.tw; 3 National Museum of Marine Biology & Aquarium, Pingtung 944, Taiwan; E-Mail: x2219@nmmba.gov.tw; 4 Department of Medical Administration, Antai Medical Care Cooperation Antai Tian-Sheng Memorial Hospital, Pingtung 92842, Taiwan; E-Mail: weitung62@yahoo.com.tw; 5 Department of Biological Science and Technology, Meiho University, Pingtung 91202, Taiwan

**Keywords:** spatane diterpenoid, soft coral, *Sinularia leptoclados*

## Abstract

A new spatane diterpenoid, leptoclalin A (**1**), along with two previously reported known norcembranoid diterpenes (**2** and **3**), were isolated from a cultured soft coral *Sinularia leptoclados.* The structures were determined by extensive spectroscopic analyses and by comparison with the spectral data of related known compounds. Metabolite **1** is rarely found in spatane skeletons reported from soft corals. In addition, compound **1** exhibited weak cytotoxicity towards human tumor cell lines T-47 D and K-562.

## 1. Introduction

**Figure 1:**
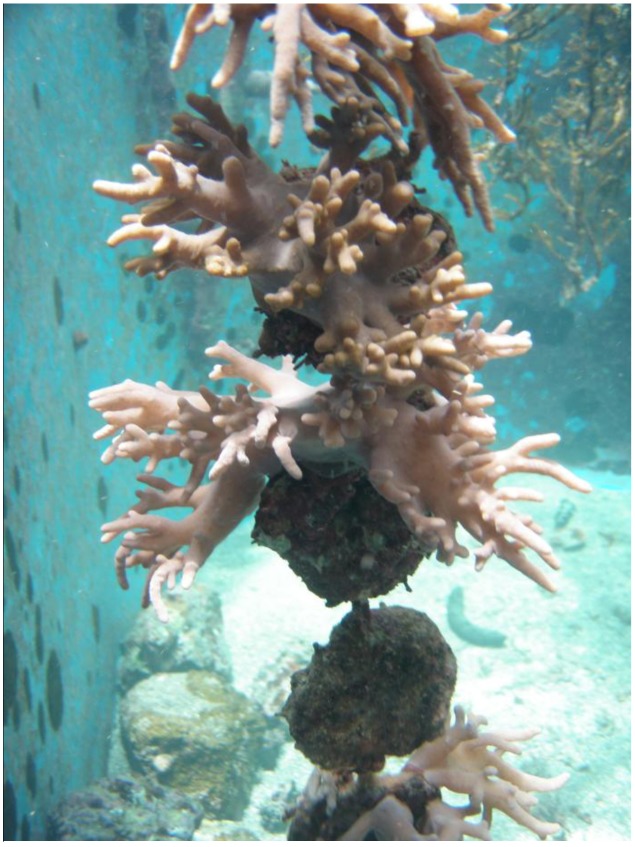
Soft coral *Sinularia leptoclados*.

**Chart 1:**
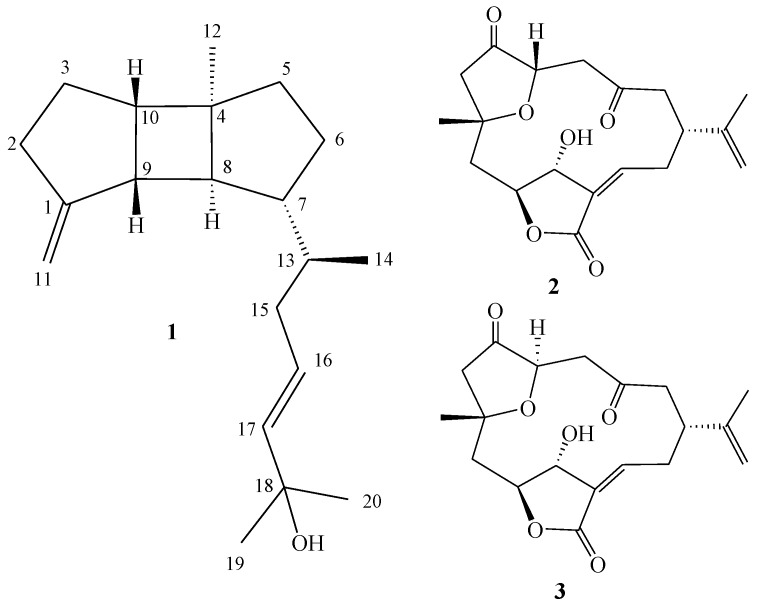
Structures of metabolites **1**–**3**.

In previous studies, soft corals have emerged as one of the most prolific sources of novel secondary metabolites [1]. Some of these have exhibited various biological activities, such as cytotoxic [2,3], anti-inflammatory [4,5,6], antiviral [6] and antifouling [7] activities. However, the bioactive secondary metabolites contents of wild soft coral are small, and extraction of large amounts of bioactive metabolites from wild coral is not feasible. Therefore, scientists are searching for new ways to obtain large sources of bioactive metabolites. In recent years, the farming techniques of soft corals have improved considerably. Therefore, researchers have been able to obtain greater amounts of soft corals, and thus larger amounts of bioactive metabolites, and have been engaged in examining the various pharmacological activities. During the course of our group’s (National Museum of Marine Biology & Aquarium, Taiwan) search for bioactive metabolites from cultured soft corals, several diterpenoids have been isolated from the soft corals *Klyxum simplex* [8,9,10,11], *Sinularia flexibilis* [12], *Sarcophyton trocheliophorum * [13], and *Lobophytum crassum * [14]*.* In continuation of our search for biologically active secondary metabolites from the cultured soft coral *Sinularia leptoclados *(Figure 1), we have isolated one new spatane diterpenoid, leptoclalin A (**1**), along with two known norcembranoid diterpenes, 5-episinuleptolide (**2**) and sinuleptolide (**3**) [15] (Chart 1). The structure of **1** was established by detailed spectroscopic analysis, including extensive examination of 2D NMR (^1^H–^1^H COSY, HMQC and HMBC) correlations. The cytotoxicity of compounds **1**–**3** against four cancer cells, DLD-1 (human colon adenocarcinoma), HCT 116 (human colorectal carcinoma), T-47D (hormone-dependent breast cancer) and K-562 (human chronic myelogenous leukemia) was studied.

## 2. Results and Discussion

The EtOAc extract of the freeze-dried specimen was fractionated by silica gel column chromatography and the eluted fractions were further separated utilizing normal phase HPLC to yield metabolites **1**–**3**. The new compound was given the trivial name leptoclalin A (**1**). The known compounds were identified as 5-episinuleptolide (**2**) and sinuleptolide (**3**). 

Leptoclalin A (**1**) was isolated as a colorless oil. Its molecular formula was determined to be C_20_H_32_O on the basis of HR-ESI-MS (*m/z* 311.2353 [M + Na]^+^), implying five degrees of unsaturation. The IR spectrum of **1 **revealed the presence of a hydroxy functionality (ν_max_ = 3365 cm^−1^). The ^13^C-NMR and DEPT spectroscopic data (Table 1) of **1** indicated the presence of four methyls, six methylenes, seven methines, and three quaternary carbons, implying, from the required degrees of unsaturation, a tricyclic diterpene framework. The NMR signal at δ_C_ 70.7 (C) showed the presence of a hydroxy group. A 1,1-disubstituted and a 1,2-disubstituted double bond were also identified from NMR signals appearing at δ_C_ 157.5 (C), 103.7 (CH_2_), and δ_H_ 4.74 (1H, s) and 4.70 (1H, s), and at δ_C_ 139.2 (CH), 125.8 (CH), and δ_H_ 5.58 (2H, m), respectively. Moreover, the ^1^H NMR spectroscopic data of **1** revealed evidence of four methyl groups at δ 0.83 (3H, d, *J* = 6.5 Hz), 1.00 (3H, s) and 1.31 (6H, s).

**Figure 2:**
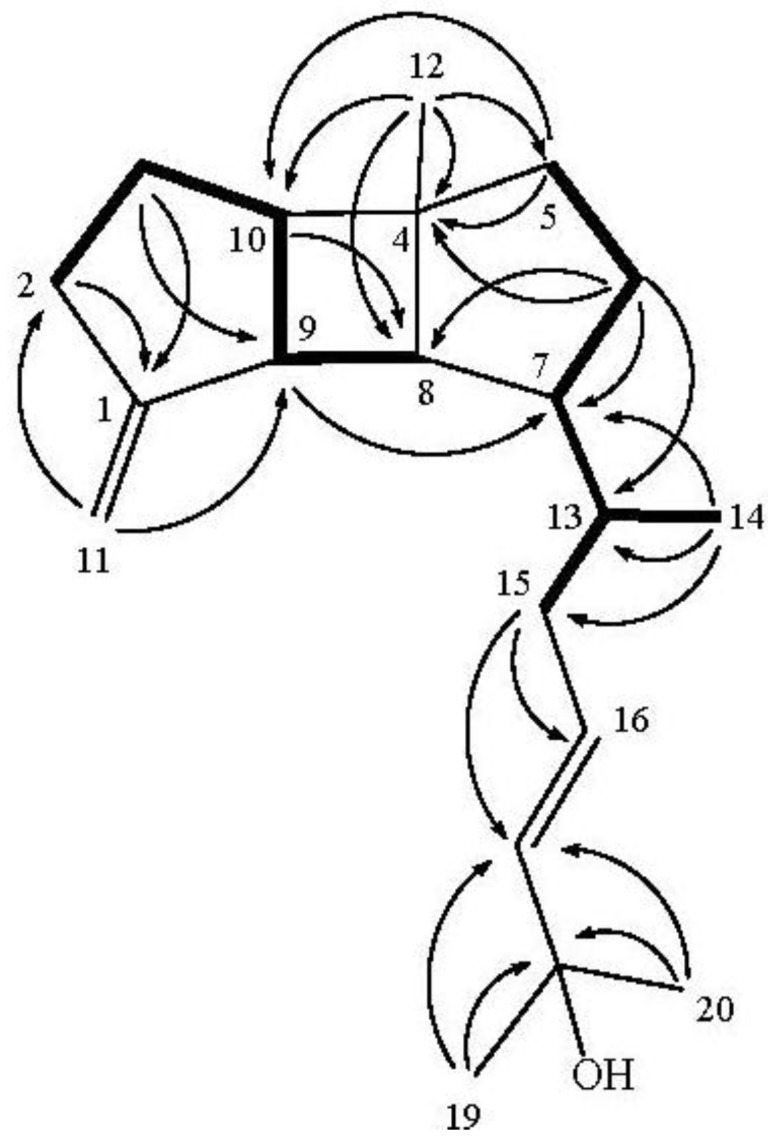
Selected ^1^H−^1^H COSY (▬) and HMBC (→) correlations of **1**.

The planar structure and all of the ^1^H and ^13^C chemical shifts of **1** were elucidated by 2D NMR experiments, in particular ^1^H–^1^H COSY and HMBC experiments (Figure 2). From the ^1^H–^1^H COSY spectrum (CDCl_3_), it was possible to establish the proton sequences from H_2_-2 to H-10 through H_2_-3; H_2_-5 to H_2_-6; H-8 to H-10 through H-9; and H-13 to H_3_-14. The overlapping of proton singals of H-7 and H-8 at δ_H_ 1.62 ppm, measured in CDCl_3_, was clearly resolved by measuring the ^1^H NMR spectrum in pyridine-*d*_5_ (see Table 1) into two separate protons at δ_H_1.62 and 1.67, respectively. ^1^H–^1^H COSY correlations (pyridine-*d*_5_) were observed between H-6 to H-13 through H-7. Therefore, the ^1^H–^1^H COSY experiment allowed the building of the two partial structures of the consecutive proton spin systems indicated in bold in Figure 2. These data, together with the HMBC correlations (Figure 2) from H_2_-2 to C-1, H_2_-3 to C-1 and C-9, H_2_-5 to C-4 and C-10, H_2_-6 to C-4 and C-8, H-9 to C-7 and H-10 to C-8 established the connectivity within the 5-membered, 4-membered and 5-membered rings. Moreover, the correlations of H_2_-11 to C-2 and C-9 indicated the attachment of an sp^2^ methylene at C-1, and the methyl was attached at C-4 on the basis of the correlations of H_3_-12 to C-4, C-5, C-8 and C-10. The planar structure of the side chain was elucidated mainly from the key HMBC correlations from H_3_-14 to C-7, C-13, and C-15, H_2_-15 to C-16 and C-17, and both methyls H_3_-19 and H_3_-20 to C-18 and C-17, and thus the connectivity from C-13 to C-20 was fully established. The above findings suggested that **1** has a tricyclic carbon skeleton. 

**Table 1:** ^1^H and ^13^C NMR data for **1**.

	δ_H_ (*J* in Hz) ^a^	δ_C_ (mult.) ^b^	δ_H_ (*J* in Hz) ^c^	δ_C_ (mult.) ^d^
1		157.5 (C)		157.8 (C)
2	2.54 m; 2.28 m	33.7 (CH_2_)	2.55 m; 2.27 m	34.4 (CH_2_)
3	1.81 m; 1.59 m	27.2 (CH_2_)	1.74 m; 1.52 m	28.0 (CH_2_)
4		43.1 (C)		43.8 (C)
5	1.50 m	42.0 (CH_2_)	1.48 m	42.7 (CH_2_)
6	1.89 m; 1.55 m	29.7 (CH_2_)	1.84 m; 1.54 m	30.6 (CH_2_)
7	1.62 m	54.5 (CH)	1.62 m	55.3 (CH)
8	1.62 m	55.0 (CH)	1.67 m	55.8 (CH)
9	2.35 m	47.8 (CH)	2.46 m	48.6 (CH)
10	2.32 m	45.7 (CH)	2.24 m	46.4 (CH)
11	4.74 s; 4.70 s	103.7 (CH_2_)	4.89 s; 4.87 s	104.8 (CH_2_)
12	1.00 s	21.6 (CH_3_)	0.94 s	22.0 (CH_3_)
13	1.20 m	36.3 (CH)	1.20 m	37.4 (CH)
14	0.83 d (6.5)	17.8 (CH_3_)	0.88 d (6.5)	18.4 (CH_3_)
15	2.15 ddd (13.5, 3.5, 3.5); 1.74 m	38.2 (CH_2_)	2.27 m; 1.85 m	39.1 (CH_2_)
16	5.58 m	125.8 (CH)	5.90 m	125.1 (CH)
17	5.58 m	139.2 (CH)	5.90 m	142.0 (CH)
18		70.7 (C)		70.1 (C)
19	1.31 s	29.9 (CH_3_)	1.53 s	31.3 (CH_3_)
20	1.31 s	29.8 (CH_3_)	1.53 s	31.2 (CH_3_)

^a^ 500 MHz in CDCl_3_; ^b^ 125 MHz in CDCl_3_; ^c^ 500 MHz in pyridine-*d*_5_; ^d^ 125 MHz in pyridine-*d*_5_.

**Figure 3:**
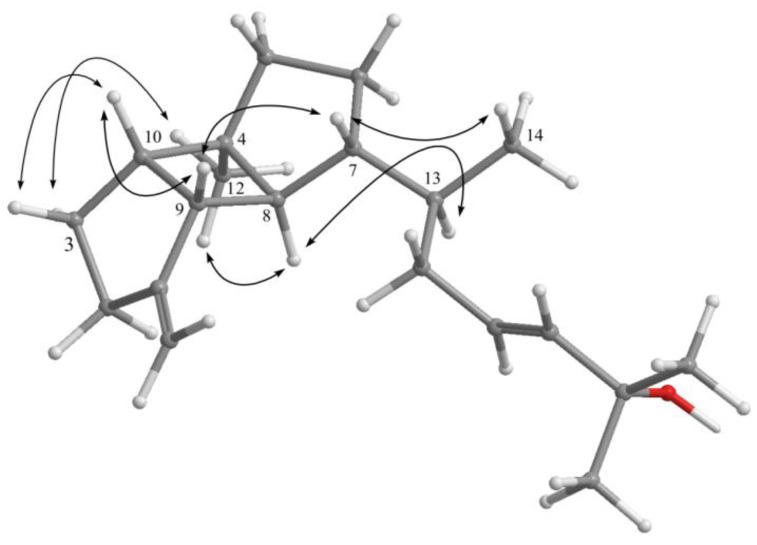
Computer-generated model of **1** using MM2 force field calculations and selected NOE correlations of **1**.

**Figure 4:**
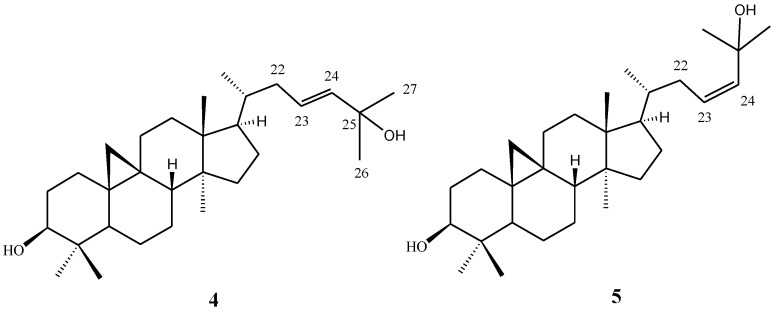
Structures of **4** and **5**.

A computer-modeled 3D structure of **1** (Figure 3) was generated using the molecular modeling program Chem3D Ultra version 9.0 and MM2 force-field calculations for energy minimization. The relative structure of **1**, assigned by the analysis of NOE correlations, was compatible with that of **1 **offered by computer modeling, in which the close contacts of the atoms in space were consistent with the NOE correlations. By the NOESY spectrum (pyridine-*d*_5_), it was found that H-9 (δ 2.46) showed NOE interactions with H-7 (δ 1.62) and H-10 (δ 2.24); therefore, assuming the β-orientation of H-9, H-7 and H-10 should also be positioned on the β face. One of the methylene protons at C-3 (δ 1.52) exhibited NOE correlations with H-10 and was characterized as H-3β, while the other (δ 1.74) was designated H-3α. NOE correlations observed between H-3α and H_3_-12 (δ 0.94) and H_3_-12 and H-8 (δ 1.67) reflected the α-orientations of H_3_-12 and H-8. Moreover, NOE correlations were observed between H-8 with H-13 but not with H_3_-14, and between H-7 with H_3_-14, indicating that H_3_-14 has a β-orientation. Furthermore, the configuration of the double bond at C-16/C-17 was determined by comparison of the NMR data of **1** in CDCl_3_ with those of two related synthetic compounds, (23*E*)-cycloart-23-ene-3β,25-diol (**4**) and (23*E*)-cycloart-23-ene-3β,25-diol (**5**) (Figure 4), also measured in CDCl_3_ (Table 2) [16]. Comparison of the NMR data of **1** and **4 **confirmed that both compounds have the same partial structure from C-15 to C-20 of **1** and from C-22 to C-27 of **4**. Furthermore, the geometry at C-16 double bond is deducible with the ^13^C chemical shift of C-15 methylene (the downfield chemical shift at 38.2 ppm clearly indicates the *E* geometry). Thus, it was suggested that the geometry of **1** at C-16/C-17 is *E*. On the basis of the above analysis, the structure of **1** was established.

**Table 2:** Selective ^1^H and ^13^C NMR data for **4** and **5** ^a^.

	4 ^b^		5 ^b^	
position	δ_H_ (*J* in Hz)	δ_C_ (mult.)	δ_H_ (*J* in Hz)	δ_C_ (mult.)
22a	1.74 m	39.0 (CH_2_)	2.13 dddd (14.8, 9.9, 8.8, 1.7)	34.6 (CH_2_)
22b	2.17 ddd (14.0, 3.3, 3.3)		2.38 dddd (14.8, 6.1, 3.8, 1.7)	
23	5.60 m	125.6 (CH)	5.31 ddd (12.1, 8.8, 6.1)	130.2 (CH)
24	5.60 m	139.4 (CH)	5.53 ddd (12.1, 1.7, 1.7)	137.4 (CH)
25		70.7 (C)		71.6 (C)
26	1.31 s	30.0 (CH_3_)	1.36 s	31.3 (CH_3_)
27	1.32 s	29.9 (CH_3_)	1.37 s	31.1 (CH_3_)

^a^ Reference 10; ^b^ NMR data in CDCl_3_.

Finally, we used the 3-(4,5-dimethylthiazol-2-yl)-2,5-diphenyl tetrazolium bromide (MTT) assay to examine the cytotoxic activities of compounds **1**–**3** against four cancer cells, DLD-1 (human colon adenocarcinoma), HCT 116 (human colorectal carcinoma), T-47D (hormone-dependent breast cancer), and K-562 (human chronic myelogenous leukemia). Cells were treated with different concentrations of **1**–**3** for 72 h. The results show that compound **1** was found to show weak cytotoxicity towards the growth of T-47D and K-562 tumor cells (the IC_50_ values were 15.4 and 12.8 μg/mL for T-47D and K-562, respectively) (Table 3). The other tested compounds were not cytotoxic (IC_50_ > 20 μg/mL) towards the above four cancer cell lines (Table 3).

**Table 3:** Cytotoxicity (IC_50_μg/mL) of compounds **1**–**3**.

	Cell Lines			
Compound	DLD-1	HCT-116	T-47D	K-562
1	NA ^b^	NA ^b^	15.4	12.8
2	NA ^b^	NA ^b^	NA ^b^	NA ^b^
3	NA ^b^	NA ^b^	NA ^b^	NA ^b^
Doxorubicin ^a^	0.42	0.89	0.28	0.14

^a^ Clinical anticancer drug used as a positive control; ^b^ NA, not active at 20 μg/mL.

## 3. Experimental Section

### 3.1. General Experimental Procedures

Optical rotation values were measured with a Jasco P-1010 digital polarimeter. IR spectra were recorded on a Varian Digilab FTS 1000 Fourier transform infrared spectrophotometer. The NMR spectra were recorded on a Varian Mercury Plus 400 FT-NMR (or Varian Unity INOVA 500 FT-NMR) instrument at 400 MHz (or 500 MHz) for ^1^H-NMR and 100 MHz (or 125 MHz) for ^13^C-NMR, respectively, in CDCl_3 _and pyridine-*d*_5_. ESI-MS-spectra were obtained with a Bruker APEX II mass spectrometer. Gravity column chromatography was performed on silica gel (230–400 mesh, Merck, Darmstadt, Germany). TLC was carried out on precoated Kieselgel 60 F254 (0.2 mm, Merck, Darmstadt, Germany) and spots were visualized by spraying with 10% H_2_SO_4_ solution followed by heating. High-performance liquid chromatography (HPLC) was performed using a system comprised of a Hitachi L-7100 pump and a Rheodyne 7725 injection port. A preparative normal phase column (Hibar 250 × 21.2 mm, Supelco, silica gel 60, 5 μm) was used for HPLC.

### 3.2. Animal Material

Specimens of the soft coral *Sinularia leptoclados* were collected off the coast of Pingtung, southern Taiwan, and transplanted to a 120-ton cultivating tank equipped with a flow-through sea water system in June 2005. The cultured soft coral was harvested in December 2010. A voucher specimen (specimen No. 2010CSC-2) was deposited in the National Museum of Marine Biology and Aquarium, Pingtung, Taiwan.

### 3.3. Extraction and Separation

The soft coral *Sinularia leptoclados* (12.5 kg fresh wt) was frozen for storage and then freeze dried. The freeze-dried material (3.0 kg) was minced and extracted exhaustively with EtOAc (5 × 5 L). The EtOAc extract was evaporated to yield a residue (100.5 g), which was subjected to open column chromatography on silica gel, eluting with *n*-hexane (H)–EtOAc (E) gradient and EtOAc (E)–acetone (A) gradient to give 12 fractions: Fr-1 (eluted by H–E 100:1), Fr-2 (eluted by H–E 50:1), Fr-3 (eluted by H–E 30:1), Fr-4 (eluted by H–E 20:1), Fr-5 (eluted by H–E 10:1), Fr-6 (eluted by H–E 8:1), Fr-7 (eluted by H–E 5:1), Fr-8 (eluted by H–E 3:1), Fr-9 (eluted by H–E 1:1), Fr-10 (eluted by EtOAc), Fr-11 (eluted by E–A 1:1) and Fr-12 (eluted by acetone). Fraction 5 (2.2 g) was separated by silica gel column chromatography with gradient elution (*n*-hexane–EtOAc, 10:1 to 5:1) to yield five subfractions (5A–5E). Subfraction 5B (108 mg) was subjected to normal-phase HPLC with *n*-hexane–EtOAc (10:1) elution to afford **1** (3.4 mg). Fraction 8 (6.5 g), was further separated by silica gel open column chromatography with gradient elution (*n*-hexane–EtOAc, 1:2 to 2:1) to yield seven subfractions (8A–8G). Subfraction 8C (4.5 g) was further chromatographed over silica gel using *n*-hexane–acetone (5:1) to afford **3** (750 mg) and a mixture, which was further purified by normal phase HPLC using *n*-hexane–EtOAc (3:2) to afford **3** (350 mg) and **2** (1.2 g), respectively.

Leptoclalin A (**1**): colorless oil; [α]^24^_D_ +24 (*c* 0.14, CHCl_3_); IR (neat) ν_max_ 3365, 2927, 2865, 1456, and 1374 cm^−1^; ^13^C and ^1^H NMR data, see Table 1; ESIMS *m/z* 311 [M + Na]^+^; HRESIMS *m/z* 311.2353 [M + Na]^+^ (calcd for C_20_H_32_ONa, 311.2351).

### 3.4. Cytotoxicity Testing

Cell lines were purchased from the American Type Culture Collection (ATCC). Cytotoxicity assays of compounds **1**–**3** were performed using the MTT [3-(4,5-dimethylthiazol-2-yl)-2,5-diphenyltetrazolium bromide] colorimetric method [17,18]. 

## 4. Conclusions

Spatane diterpenoids were first reported in 1980 as having been obtained from the brown alga *Spatoglossum schmittii* from the Galapagos Islands [19]. Brown algae are now known to be a rich source of novel spatane diterpenoids [20,21,22,23,24,25,26,27]. In contrast, they are generally the minor components of marine soft coral [28]. Our investigation of the chemical constituents of the soft coral *Sinularia leptoclados* led to the obtainment of one new spatane diterpenoid (**1**). Moreover, to the best of our knowledge, metabolite **1**has a spatane skeleton rarely found in soft corals.

## Supplementary Files

Supplementary File 1 Supporting Information (PDF, 782 KB)
