# Supplementary material for: A New Spatane Diterpenoid from the Cultured Soft Coral Sinularia leptoclados
Source: Mar Drugs. 2013 Jan 10;11(1):114–23. doi: 10.3390/md11010114 (PMC3564161; doi:10.3390/md11010114)

Supporting Information

Figure S1. ^{1}H NMR spectrum of **1** in $CDCl_3$ at 500 MHz. 2
Figure S2. ^{13}C NMR spectrum of **1** in $CDCl_3$ at 125 MHz. 3
Figure S3. HMQC spectrum of **1** in $CDCl_3$. 4
Figure S4. HMBC spectrum of **1** in $CDCl_3$. 5
Figure S5. ^{1}H–^{1}H COSY spectrum of **1** in $CDCl_3$. 6
Figure S6. NOESY spectrum of **1** in $CDCl_3$. 7
Figure S7. ^{1}H NMR spectrum of **1** in pyridine-d_5 at 500 MHz. 8
Figure S8. ^{13}C NMR spectrum of **1** in pyridine-d_5 at 125 MHz. 9
Figure S9. HMQC spectrum of **1** in pyridine-d_5. 10
Figure S10. HMBC spectrum of **1** in pyridine-d_5. 11
Figure S11. ^{1}H–^{1}H COSY spectrum of **1** in pyridine-d_5. 12
Figure S12. NOESY spectrum of **1** in pyridine-d_5. 13

Figure S1. ^{1}H NMR spectrum of **1** in $CDCl_3$ at 500 MHz.

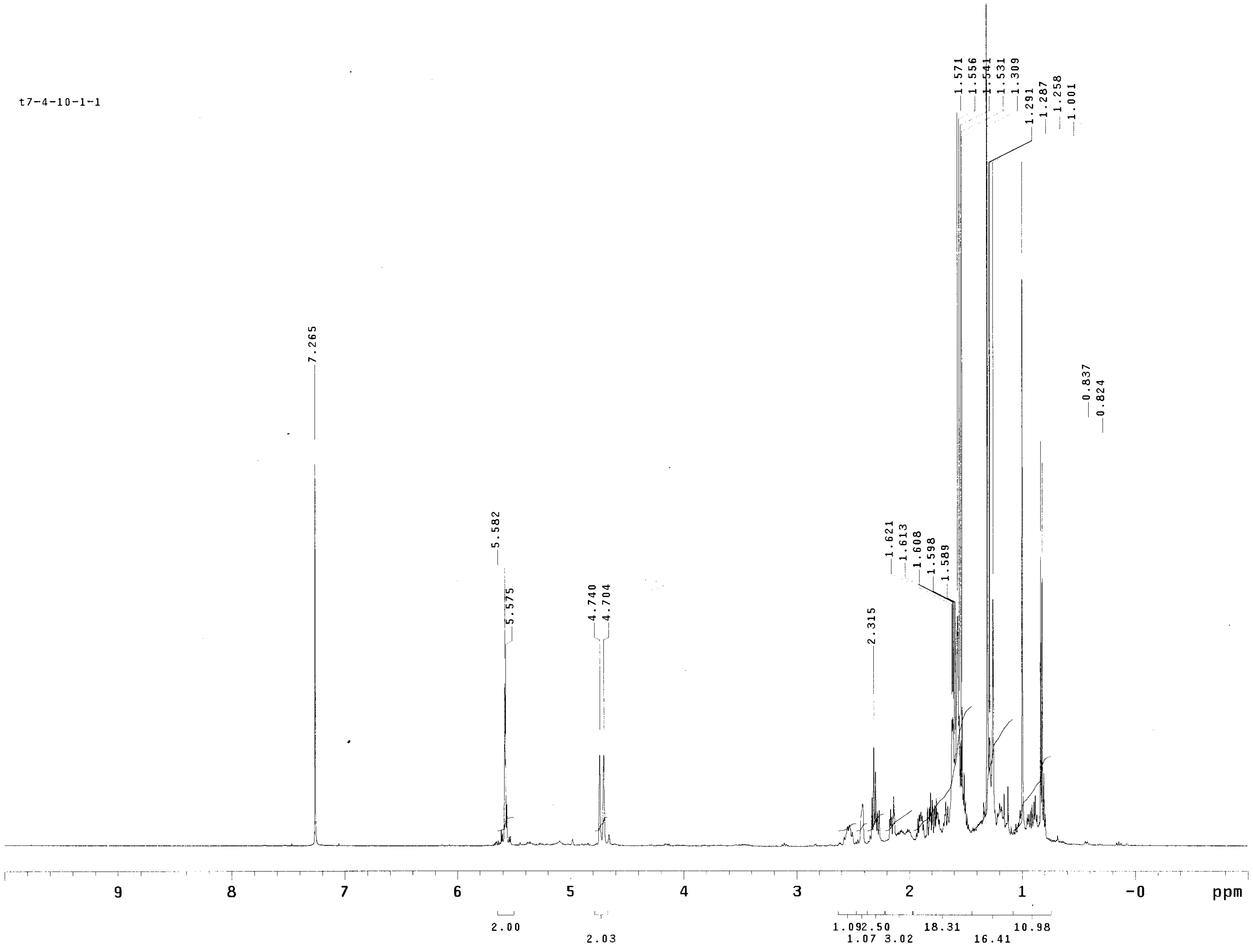

Figure S2. ^{13}C NMR spectrum of **1** in $CDCl_3$ at 125 MHz.

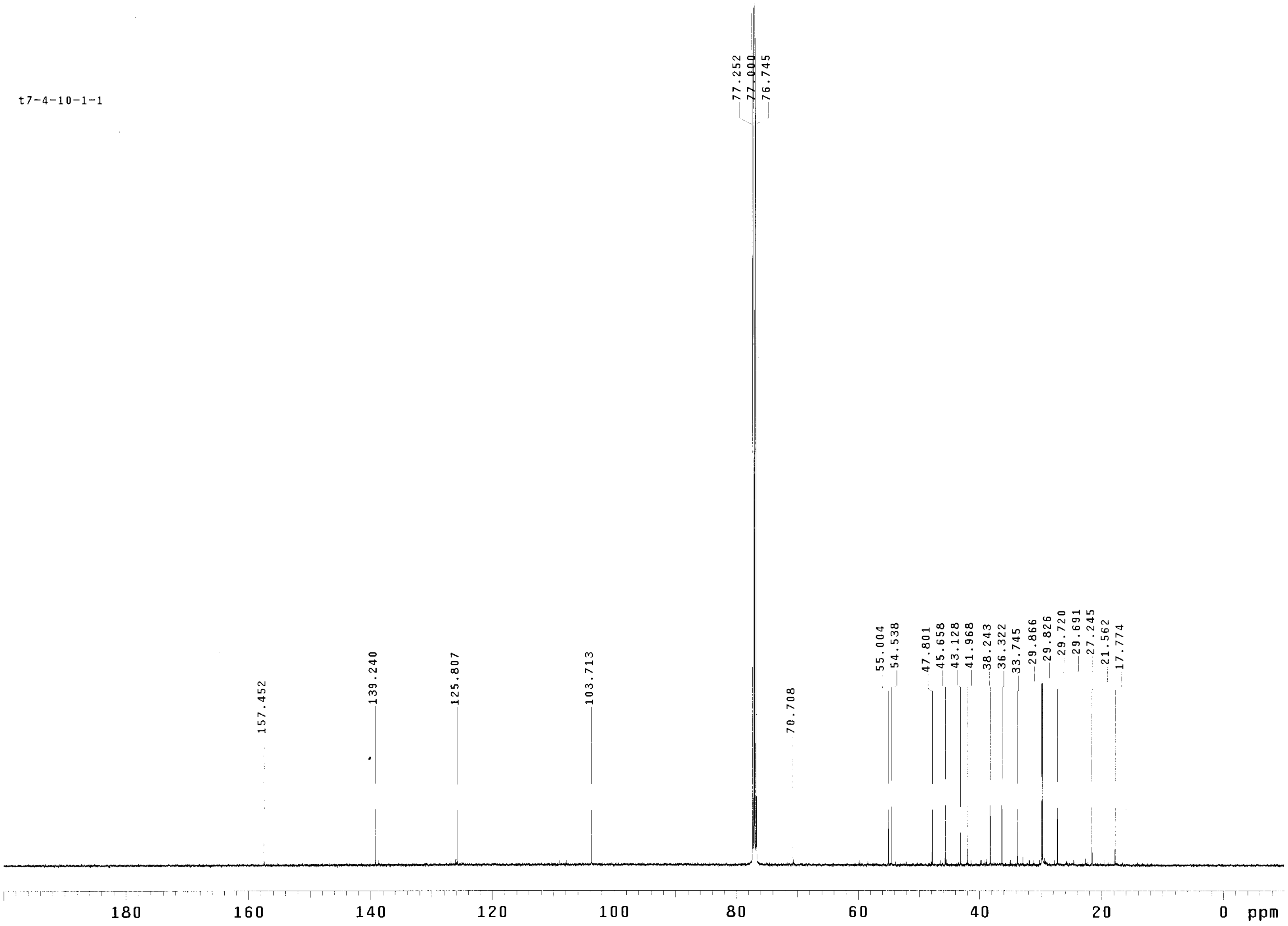

Figure S3. HMQC spectrum of **1** in $CDCl_3$.

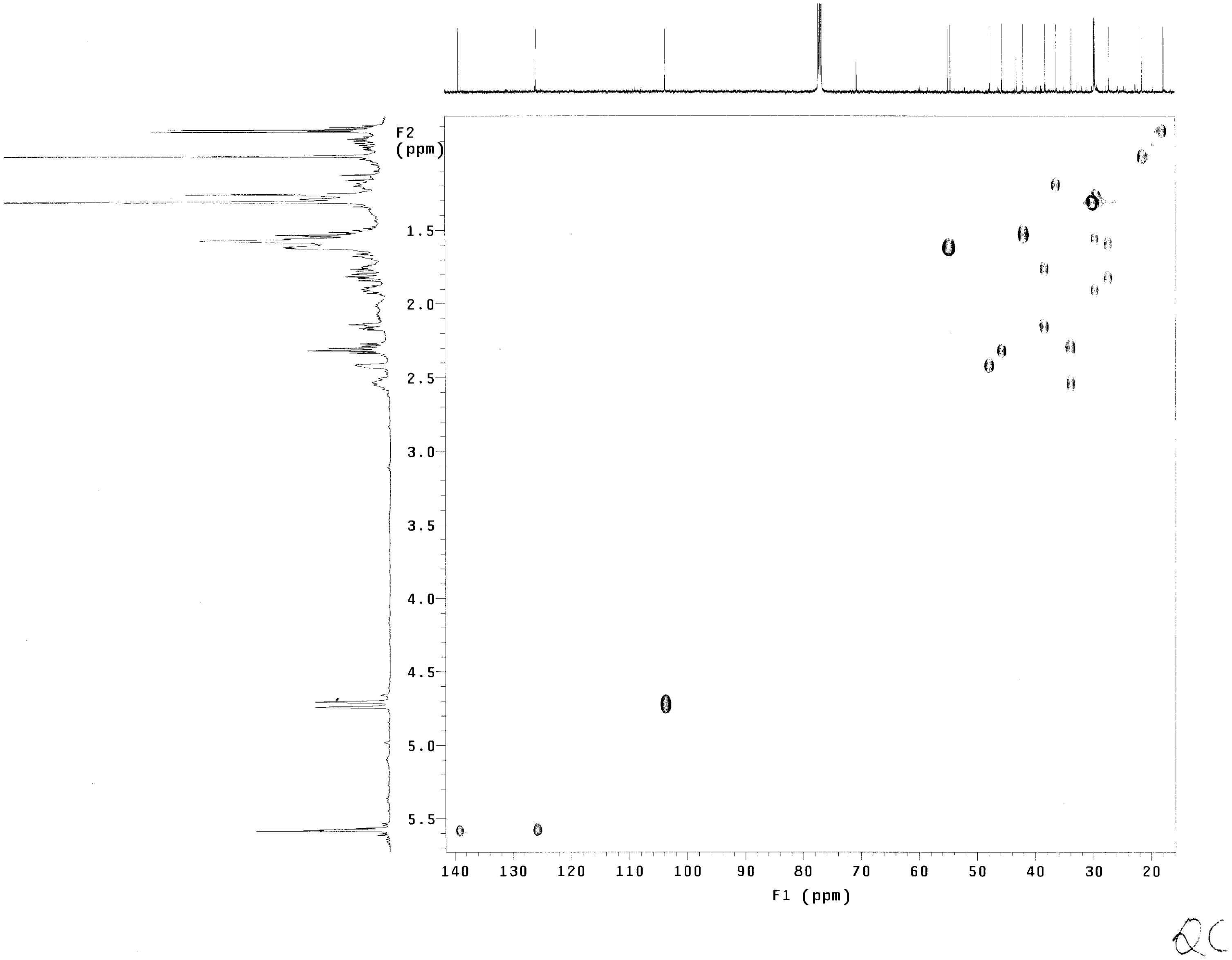

Figure S4. HMBC spectrum of **1** in $CDCl_3$.

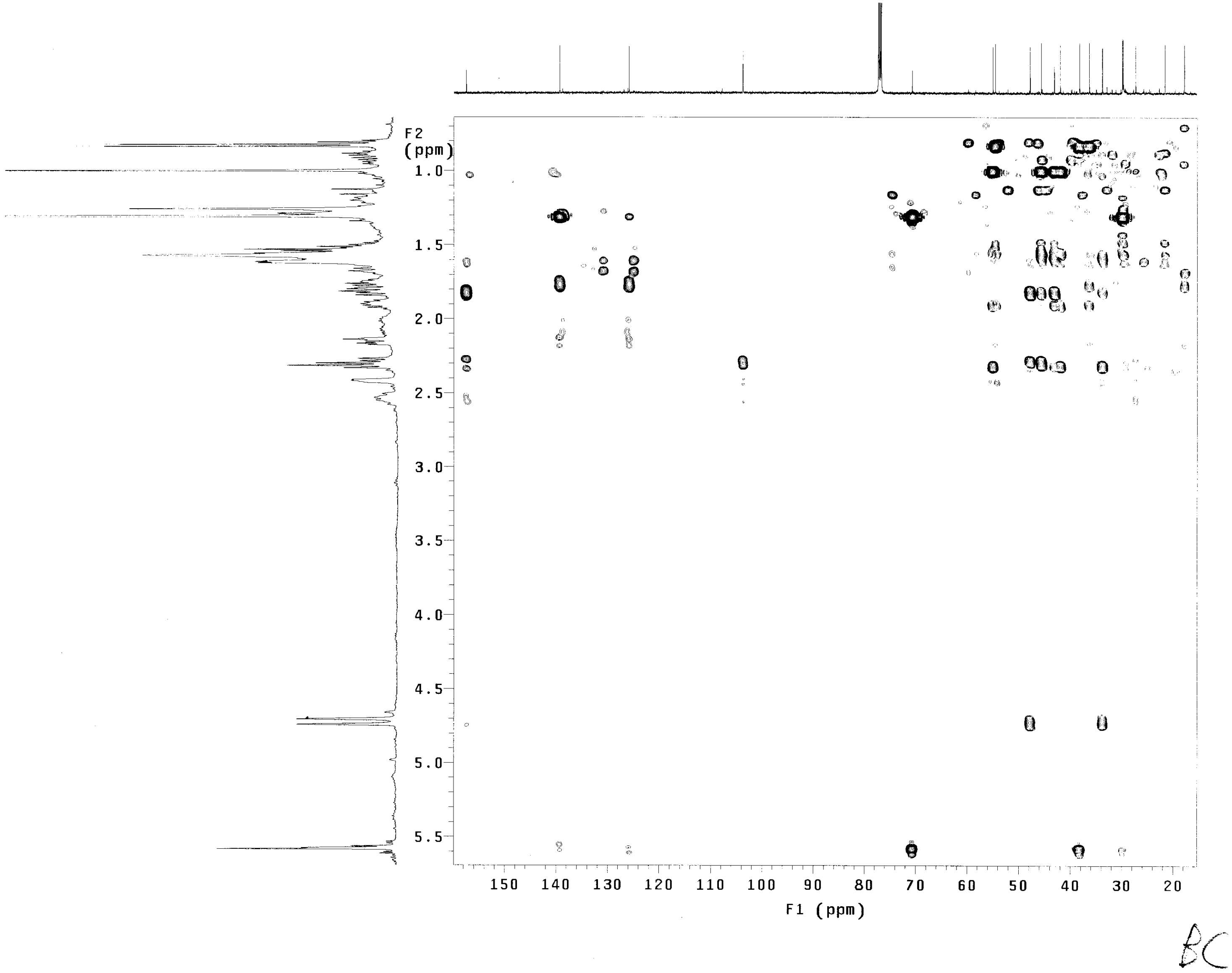

Figure S5. ^{1}H–^{1}H COSY spectrum of **1** in $CDCl_3$.

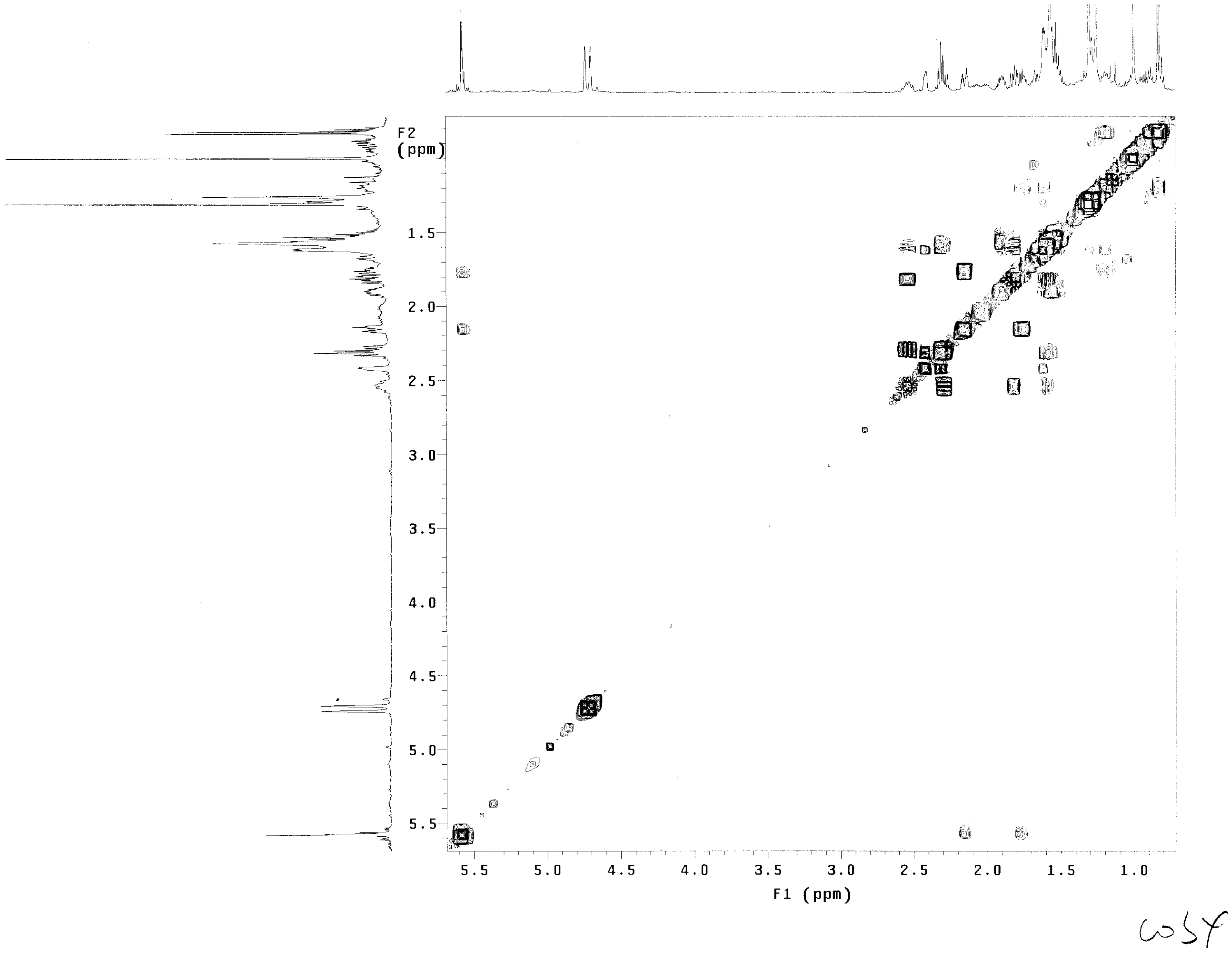

Figure S6. NOESY spectrum of **1** in $CDCl_3$.

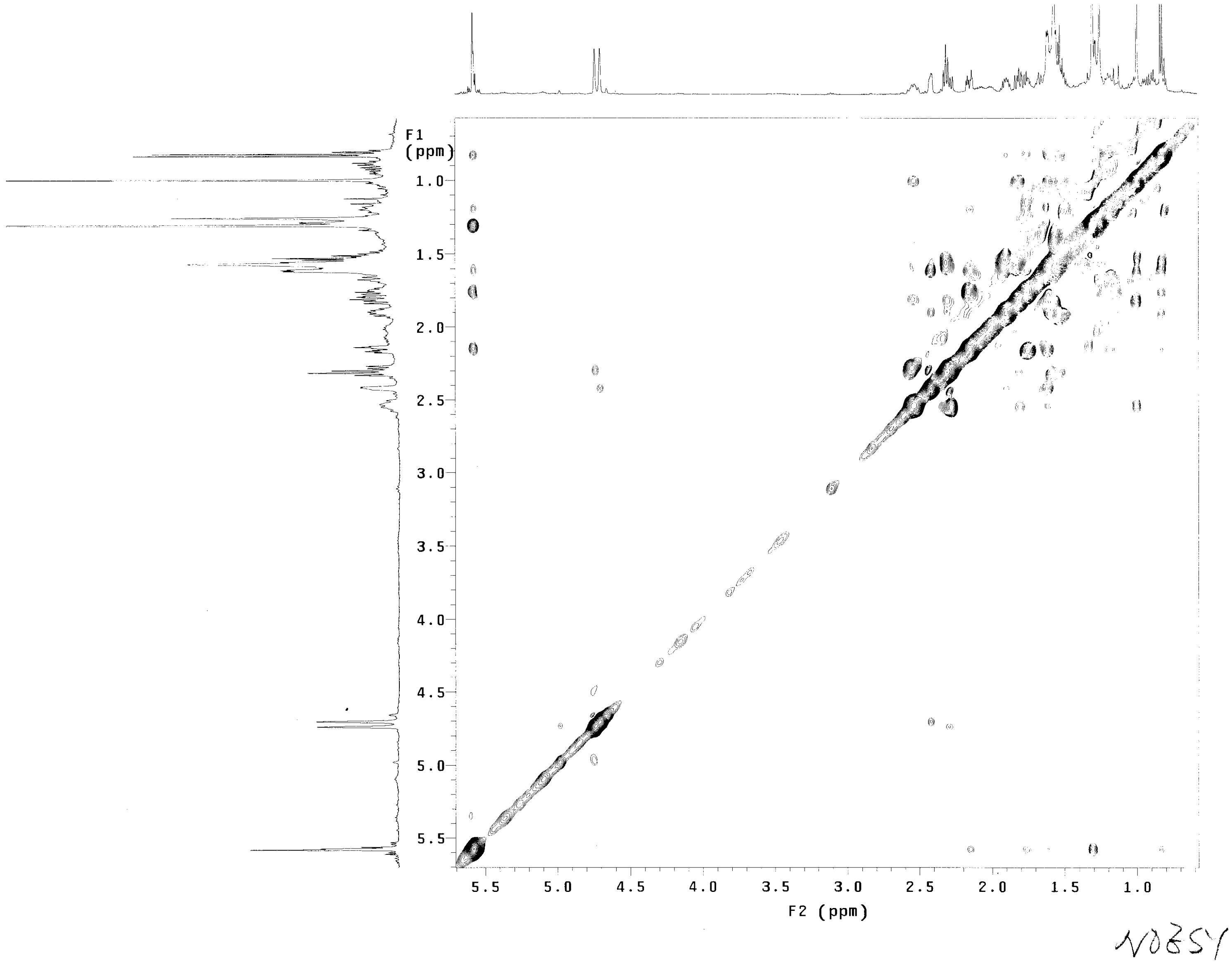

Figure S7. ^{1}H NMR spectrum of **1** in pyridine-d_5 at 500 MHz.

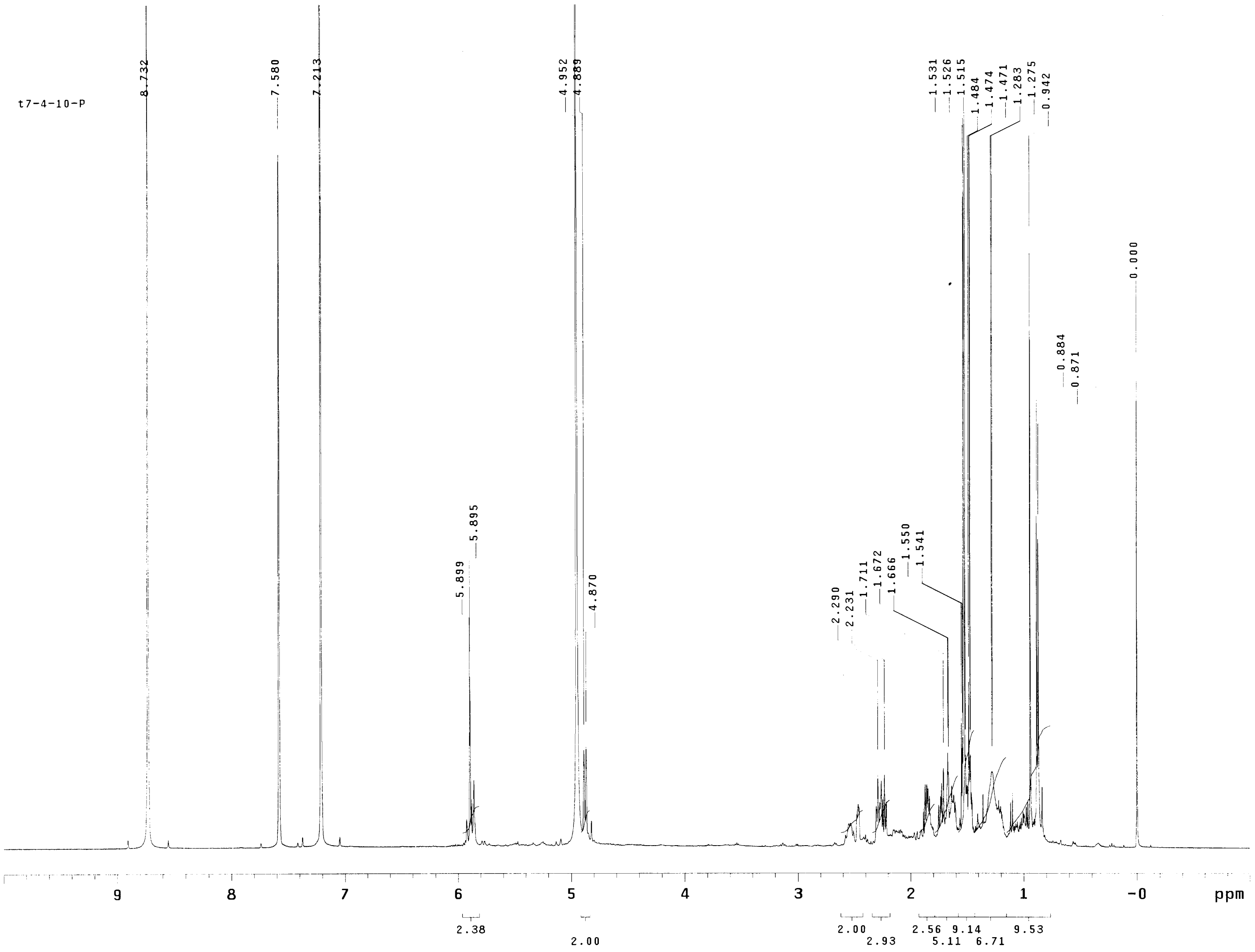

Figure S8. ^{13}C NMR spectrum of **1** in pyridine-d_5 at 125 MHz.

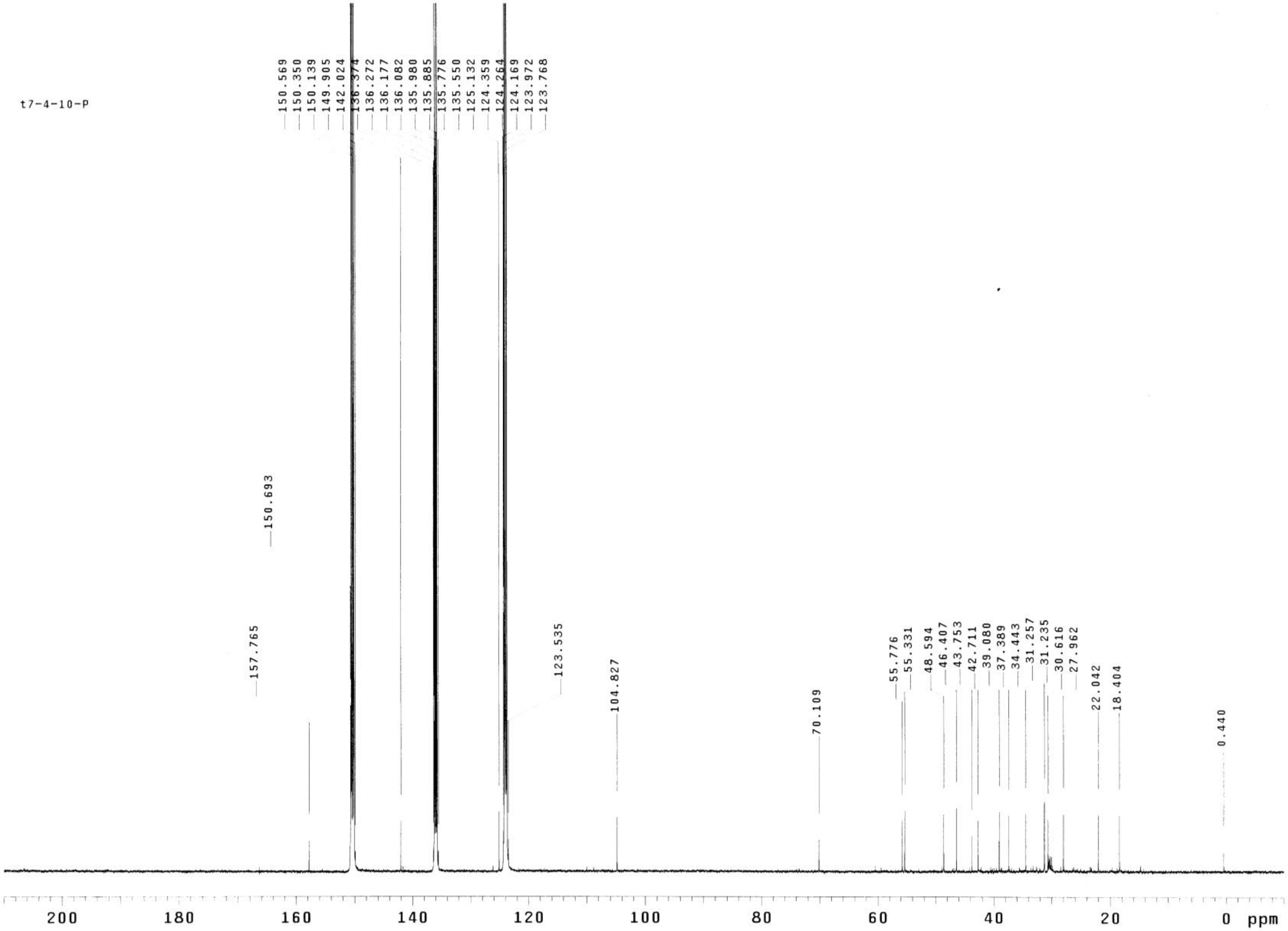

Figure S9. HMQC spectrum of **1** in pyridine-d_5.

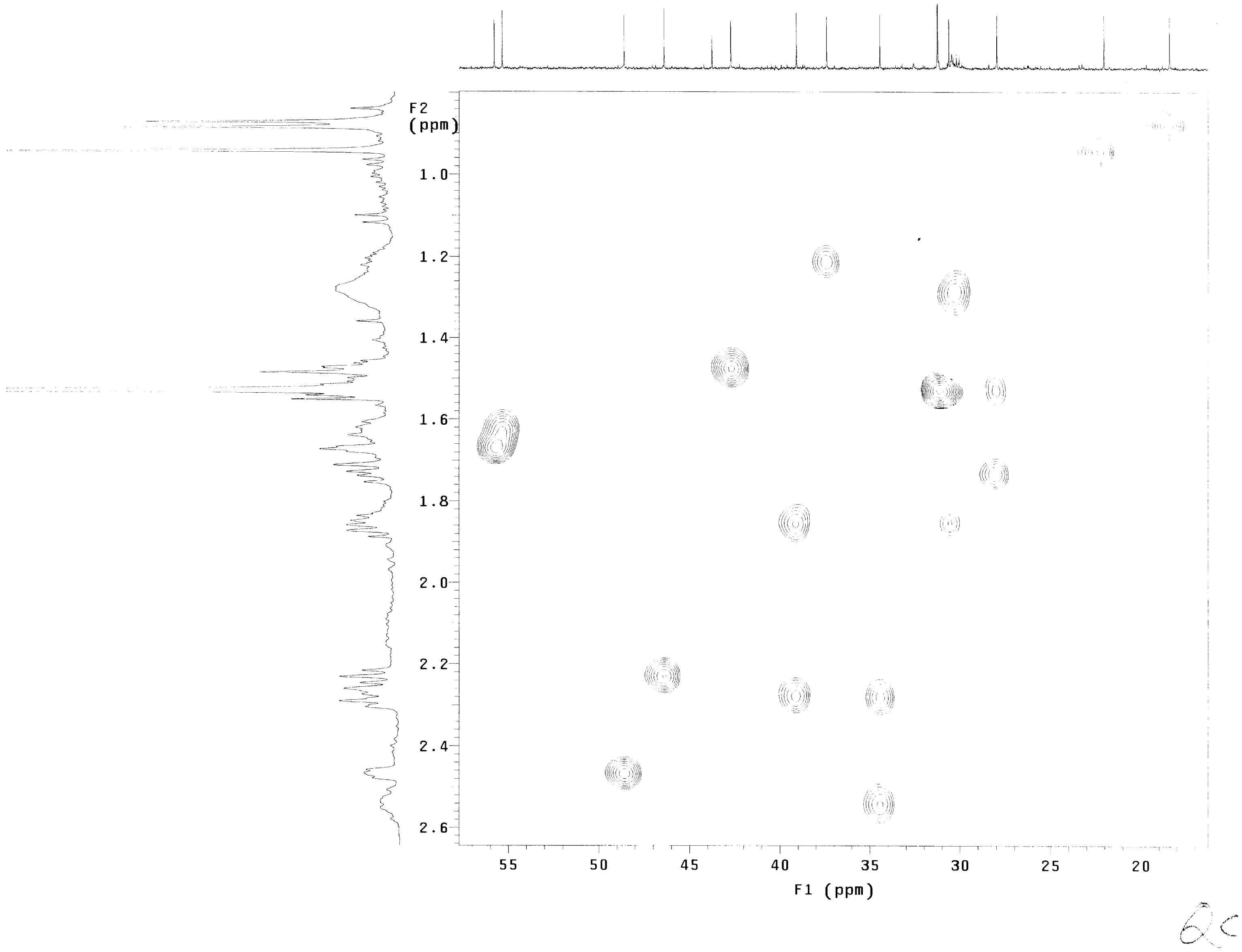

Figure S10. HMBC spectrum of **1** in pyridine-d_5.

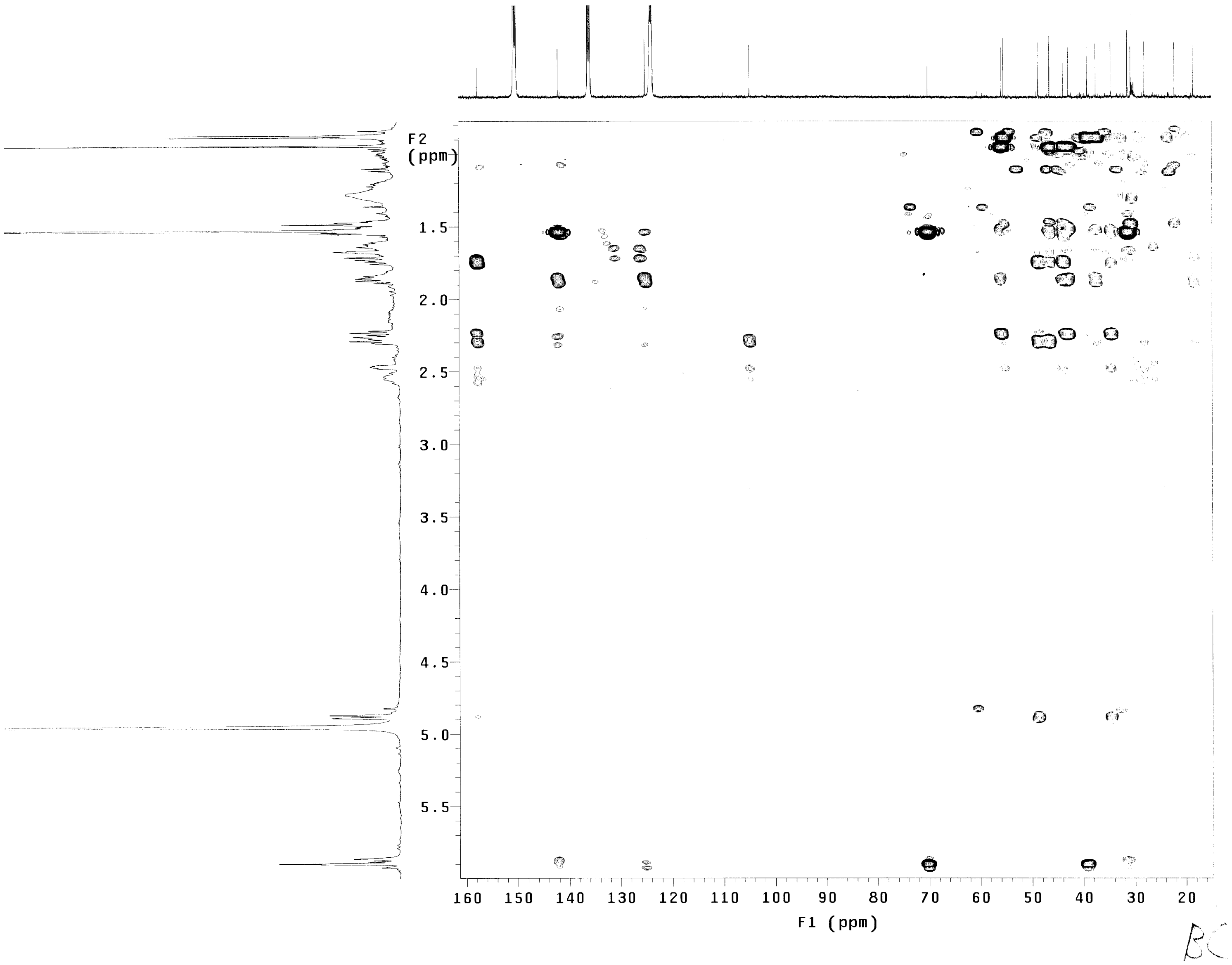

Figure S11. ^1H–^1H COSY spectrum of **1** in pyridine-d_5.

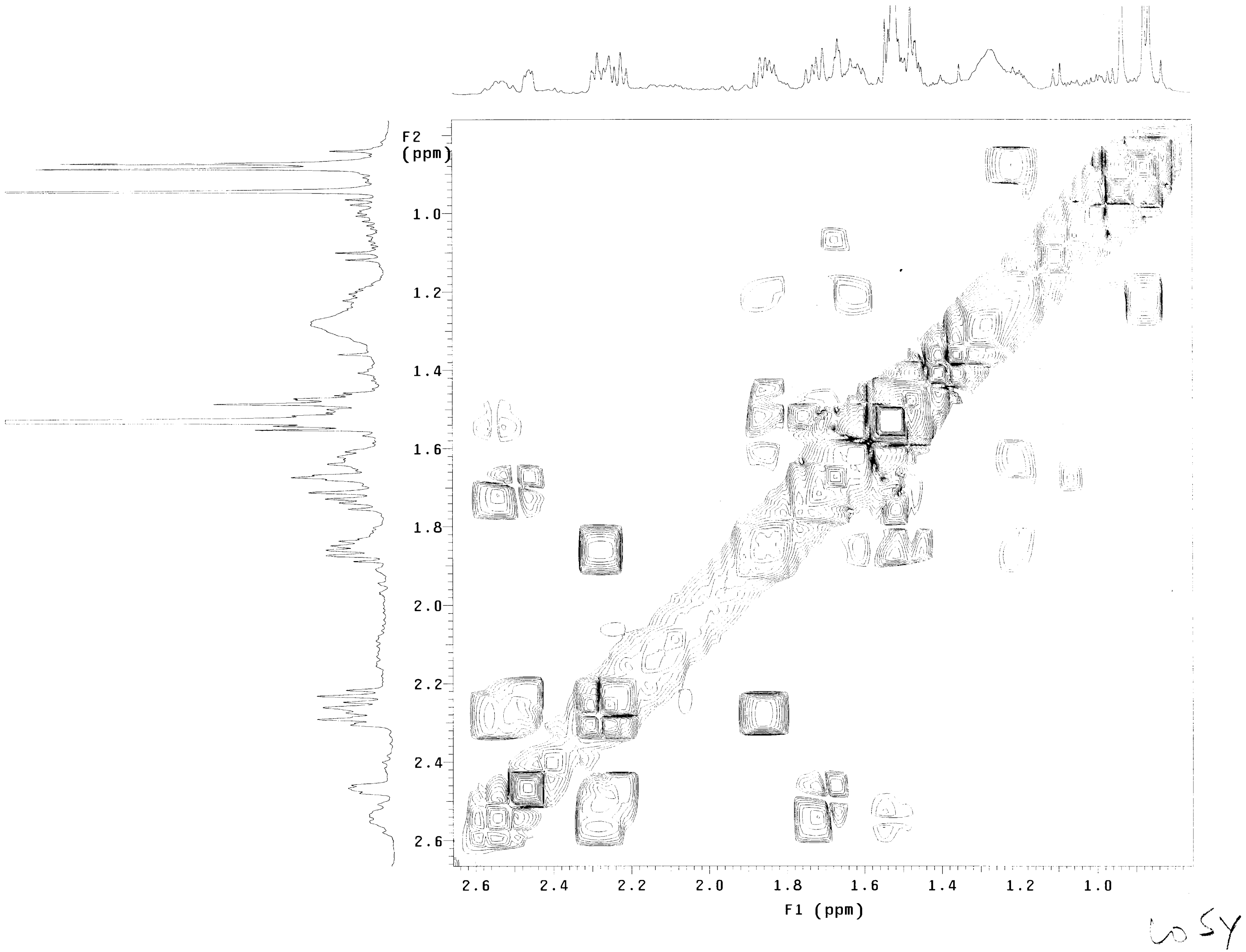

Figure S12. NOESY spectrum of **1** in pyridine-d_5.

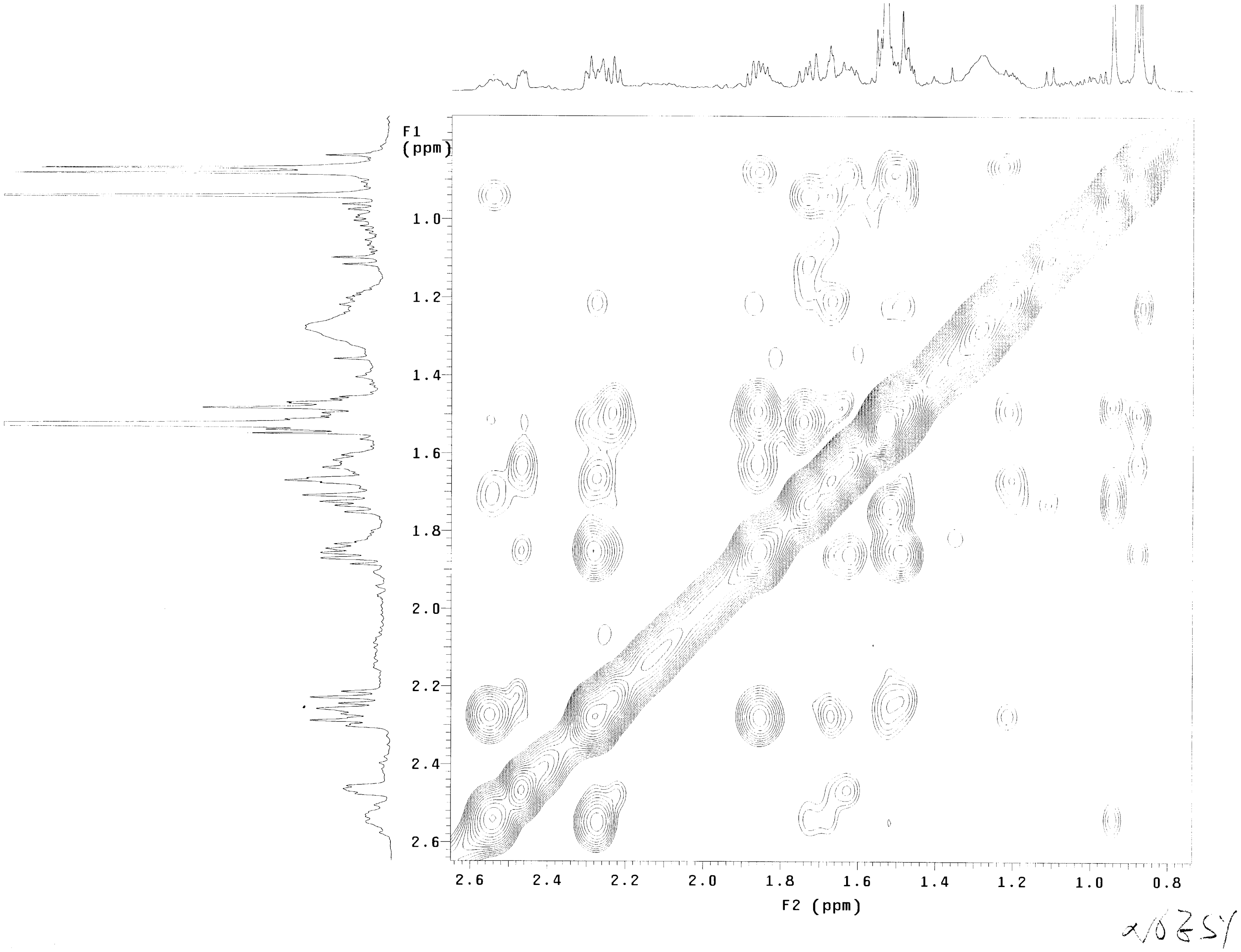

Supplement: Supplementary File 1 — Supporting Information (PDF, 782 KB) [file marinedrugs-11-00114-s001.pdf]
